# Ergonomic risk factors and musculoskeletal disorders in bank staff: an interventional follow-up study in Iran

**DOI:** 10.1186/s42506-021-00097-8

**Published:** 2021-12-11

**Authors:** Majid Motamedzadeh, Mahdi Jalali, Rostam Golmohammadi, Javad Faradmal, Hamid Reza Zakeri, Iman Nasiri

**Affiliations:** 1 Department of Ergonomics, School of Health and Research Center for Health Sciences, Hamadan University of Medical Sciences, Hamadan, Iran; 2 Department of Occupational Health Engineering, School of Health, Neyshabur University of Medical Sciences, Neyshabur, Iran; 3 Department of Occupational Health Engineering, School of Health, Isfahan University of Medical Sciences, Isfahan, Iran; 4 Center of Excellence for Occupational Health, Occupational Health & Safety Research Center, Hamadan University of Medical Sciences, Hamadan, Iran; 5 Modeling of Noncommunicable Diseases Research Center & Department of Biostatistics and Epidemiology, School of Public Health, Hamadan University of Medical Sciences, Hamadan, Iran; 6 Ferdows School of Paramedical and Health, Birjand University of Medical Sciences, Birjand, Iran; 7 Department of Ergonomics, Health Sciences Research Center, School of Health, Hamadan University of Medical Sciences, P.O. Box 65175-4171, Hamadan, Iran

**Keywords:** Bank staff, Educational intervention, Physical intervention, Musculoskeletal disorders, Rapid Office Strain Assessment (ROSA)

## Abstract

**Background:**

Long-term use of computer in a static mode may cause musculoskeletal disorders (MSDs) in bank staff. Considering the high number of bank employees in different countries, such as Iran, the risk factors of these disorders should be investigated in order to implement interventions required to reduce the risk factors. This study aimed to examine the risk factors of MSDs using the Rapid Office Strain Assessment (ROSA) method and to perform an ergonomic intervention program with banking staff in Iran.

**Methods:**

This interventional study was conducted on 277 bank employees in Iran. Subjects were randomly divided into three groups, including a control group (without any intervention), an educational intervention (EI) group, and a group receiving both educational and physical intervention (EPI). Before and after the intervention, the ROSA method and Nordic questionnaire were used to assess the risk factors of MSDs in office jobs and to investigate the prevalence of MSDs. Data were collected 2 weeks before and 9 months following the intervention.

**Results:**

Before the intervention, the mean score of ROSA at workstations of all groups was above five with high risk. Nine months after the start of the intervention, there was a significant decrease in the mean ROSA score and its components in the two groups that received the intervention, which was statistically significant (*P* < 0.001). The results of the study of the prevalence of MSDs in the employees—before the intervention—indicate that the highest prevalence of MSDs in the control group was in areas of the neck (67.1%), back (64.4%), and lower back (63%). In the EI group, the highest prevalence of MSDs was in the neck (65.2%), lower back (61.6%), and back (60.7%) areas. In the EPI group, the discomfort areas were the neck (68.5%), shoulders (66.3%), and lower back (60.9%). Nine months after the intervention, there was a significant decrease in the prevalence of MSDs in the neck, shoulders, and lumbar regions of staff who received the intervention (*P* < 0.05).

**Conclusion:**

Nine months after performing the interventions, there was a relative improvement in workstations and prevalence of MSDs in various areas within the bodies of the bank staff. This study showed that using the ROSA method is appropriate for assessing the risk factors of office work and that it can identify deficiencies in workstations. These defects can be addressed by designing and implementing an EI program together with physical interventions according to the components of the ROSA method.

## Introduction

People in their workplace routinely deal with a range of harmful occupational factors including chemicals (dust, gases, and vapors), physical hazards (noise, ionizing radiation, and inappropriate weather conditions), and psychologic and ergonomic factors (improper posture, stress, and high mental workload) [1–4]. Exposure to these factors can cause a variety of occupational complications and diseases such as respiratory diseases, musculoskeletal disorders (MSDs), physiological disorders, and cancer [1, 5, 6].

MSDs are among the most important causes of occupational injury and disability in many industries in both developed and developing countries. This results in high economic costs for these industries [7]. Currently, controlling and reducing MSDs in the workforce is one of the most important global concerns for experts in ergonomics. The importance of controlling and reducing these disorders is so high that many countries consider the prevention of work-related MSDs among the workforce as one of the national priorities [8–10]. According to surveys by the WHO, as well as the documentations provided by this organization in 2013, work-related MSDs are at second place of occupational diseases following occupational respiratory diseases [11].

The most common equipment used in most workplaces, especially banks, is the computer. This has increased exponentially over the last 20 years [12–14]. According to a report by the National Bureau of Statistics of China, in 2007, 60% of workers in the workplace use computers. This increases to 88% in business and economic services [15]. More than half of employees in European Union member countries use computers during their workday [16]. Numerous studies have shown that computer use is associated with an increase in the prevalence of MSDs. The results of longitudinal follow-up studies of 3 months to 5 years indicate an increased risk of pain in the neck and shoulders among computer users [17, 18]. Giahi et al. (2014)—in a study on bank users in Iran—showed that 70.2% of subjects had discomfort in at least one area of the body. The duration of working with a computer and inadequate resting time were the most important factors contributing to discomfort [19].

The most important physical risk factors that cause MSDs in many occupations include repetitive activity, excessive force, improper posture, contact pressures, vibration, and physical fatigue [20]. Additionally, factors such as age, sex, obesity, physical activity, and smoking (as individual factors); factors associated with workstation design such as duration of computer use, frequency of rest, keyboard usage, status of the computer monitor, and the type and use of computer-connected devices; and psychosocial factors have also been implicated in the development of MSDs [13].

Although there is growing interest among employers to improve office workplaces, few studies have examined the effects of ergonomic interventions on employees’ health. However, recent evidence suggests that ergonomic training and ergonomic design of workstations and office buildings can be useful in preventing and reducing MSDs and their associated symptoms in office settings [21]. There are various ways to reduce or eliminate the risk factors of MSDs in the office environment. The ergonomic design of the office workstations is the most effective intervention method to completely eliminate the risk factors of the office environment. However, this method is costly and time consuming. Therefore, the most sensible approach is to provide training to employees and the correct context for their own workplace settings [13]. However, there may be reasons for the inability of staff to adjust their workspaces, such as using the unadjusted equipment, workspace constraints, lack of ergonomic equipment, and a lack of ergonomic information provided to the office workers. Consequently, using the educational intervention (EI) along with physical intervention (PI) can be considered the most effective measure in improving the ergonomic conditions of office workstations. This can aid the elimination reduction of the ergonomic risk factors among office workers [22]. The present study aims to (1) assess the ergonomic risk factors of office workstations using the ROSA tool, (2) determine the effect of office ergonomics training on the improvement of workstations by staff, and (3) perform PIs to improve risk factors.

## Methods

### Study design, population, and sample

This interventional study was conducted on 277 office workers at a large bank in Iran. The participants were divided into three groups including the control group, EI group, and educational and physical intervention (EPI) group. The criteria for selecting the subjects included staff with office jobs who work with computers for at least 3 h or more per day with work experience of at least 1 year. Exclusion criteria also included refusing of participants during the study and non-occupational MSDs (due to an accident) during the study. Consequently, at the beginning of the study, of 1050 bank staff, 110 participants were selected for each group (330 people in total) using a systematic random sampling method. This study was approved by the Ethics Committee of the Hamadan University of Medical Sciences, Hamadan, Iran. All of the participants completed the informed consent and signed it.

### Data collection

After specifying the samples, the study was conducted in two phases as follows.

#### Phase I: assessment of the work environment

##### Collection of demographic and occupational data

For this purpose, a demographic characteristic questionnaire that was designed including the variables of age, sex, height, weight, and work experience was used.

##### Assessing the prevalence of MSDs

**Fig. 1:**
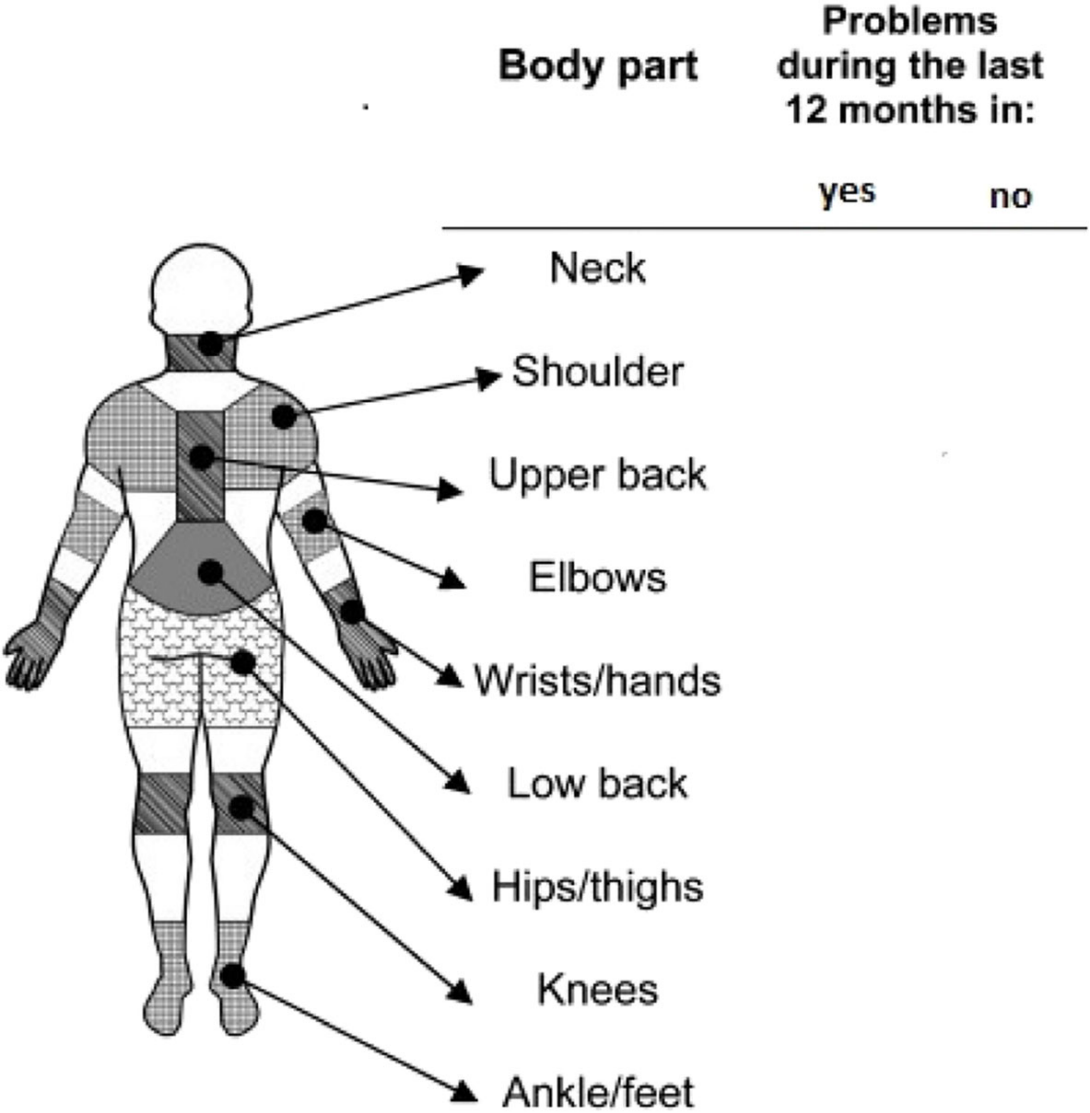
NORDIC questionnaire

The Nordic musculoskeletal questionnaire was used to determine the prevalence of MSDs before and after interventions [23]. The questionnaire consists of two general and specific sections, of which only the general section was considered based on the purpose of the study. The questionnaire was completed through direct interviews with the subjects, and the prevalence of MSDs over the year was recorded. Figure 1 presents this questionnaire.

A total of 330 questionnaires were distributed to all subjects 2 weeks before the intervention, as follows:

A total of 110 questionnaires were distributed among the 110 employees selected in the control group. Following the preintervention phase, 90 questionnaires were completed (81.8% response rate). After 9 months of intervention, the questionnaires were redistributed. This time, only 73 members of the control group completed the questionnaire (81.1% response rate). Consequently, in this group, 37 cases were excluded—compared with the beginning of the study—and 73 cases were evaluated as the final number of the control group.

A total of 120 questionnaires were distributed among 120 employees present in the EI group. At the conclusion of the preintervention phase, 112 questionnaires were completed (93.3% response rate). After 9 months of intervention, the questionnaires were redistributed, and all of 112 participants completed the questionnaire (100% response rate). Consequently, eight staff were excluded from the study—compared with the beginning of the study—and 112 staff were studied as the final number of the EI group.

**Fig. 2:**
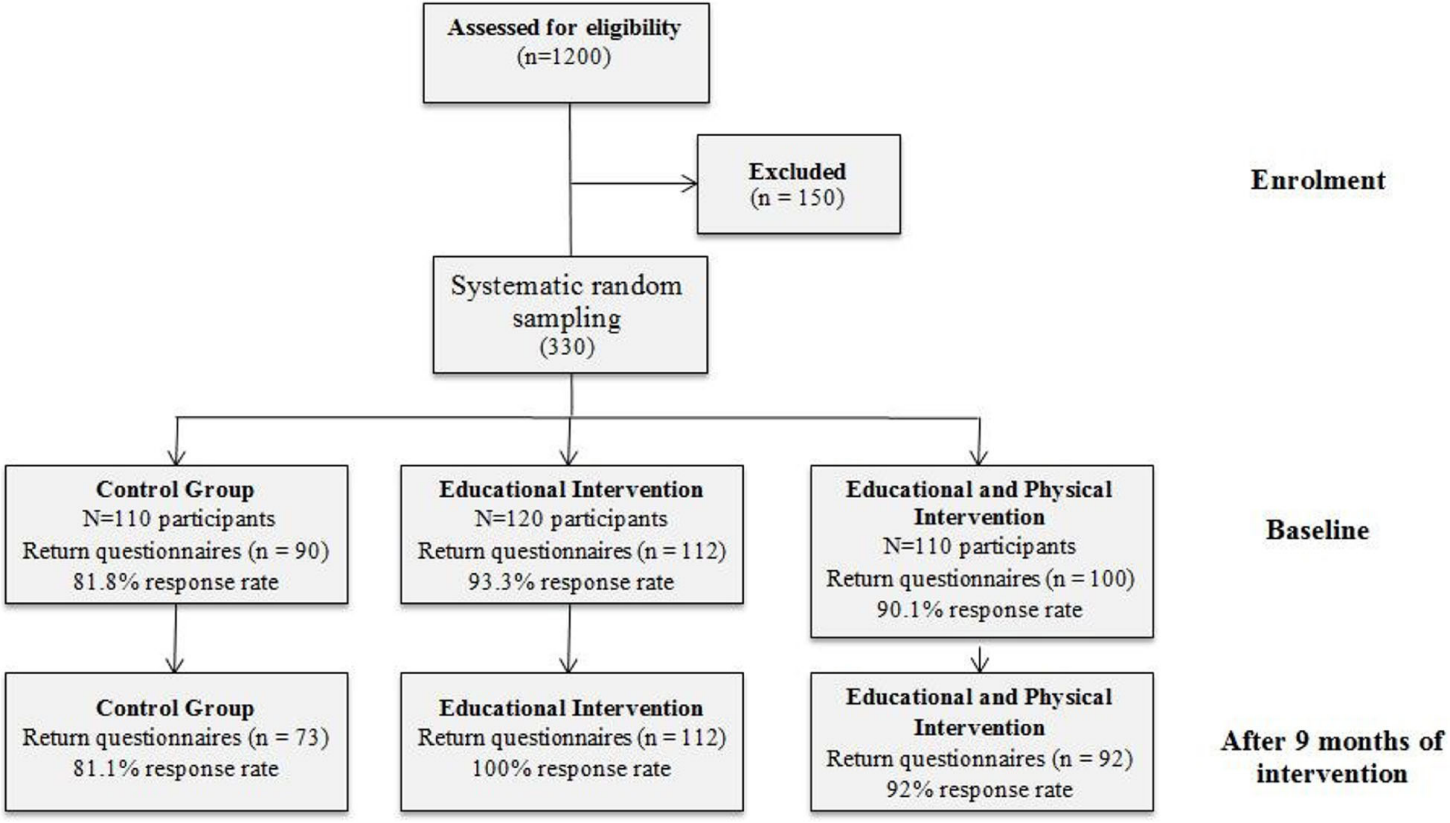
Study flow diagram

A total of 110 questionnaires were distributed among 110 employees in the EPI group. At the end of the preintervention phase, 100 questionnaires were completed (90.1% response rate). After 9 months of intervention, the questionnaires were redistributed, and only 92 individuals completed the questionnaire (92% response rate). Consequently, 18 participants were excluded from the study—compared with the beginning of the study—and 92 staff were evaluated as the final number of the EPI group. Figure 2 presents the study flow diagram.

##### Workstation ergonomic risk analysis and determination of risk levels

The Rapid Office Strain Assessment (ROSA) method was used to identify the risk factors for office work and prioritize the optimal fit between staff and workstation equipment (24). This method was developed by Sonne et al. in 2011. ROSA is a pen-and-paper approach based on Canada’s CSA-Z412 standard, which divides the workstation into several segments, including chair components, monitor, telephone, mouse, and keyboard, and determines the risk level of each section. A ROSA score above five indicates that the risk level of work is high and immediate correction is required (24).

#### Phase II: intervention

At this stage, workstations were assessed using the ROSA method and risk levels were determined. Subsequently, ergonomic interventions were designed and performed according to the components of the ROSA method for the stations that were diagnosed as requiring intervention.

Interventions that included EPIs were conducted as follows:

##### Educational intervention

These interventions consisted of preparing an educational handbook with the topic of office ergonomics distributed among all participants in the EI and EPI groups. The educational content of the handbook was selected on the basis of the design of the workstation by personnel under study—according to the risk factors examined by the ROSA method—and using the office workstation standard provided by the Canadian Standard Association [30]. This handbook included how to set up and place items on the surface of the desk, identifying neutral and inappropriate postures, how to adjust chairs and create appropriate postures while working on chairs, correct posture in the use of the mouse and keyboard, how to place and set up a monitor on the work surface, the proper position of the phone in relation to the position of staff, how to use the phone properly, and the correct place for the holder (sheets holder) on the work surface. After distributing the handbooks to the staff, they were asked to adjust their workstations according to the standards provided, if possible. The researcher transmitted face-to-face training in relation to potential questions for all personnel present—in both interventional groups—to construct a workstation. Another training activity in this phase of the study involved training staff on how to perform soft movements behind their desk. To increase the effectiveness of this approach, ErgoPro software version 2.0—designed for this purpose—was installed on all employees’ systems. The software was automatically activated at different times of the day and displayed on the staff computer monitor screen, reminding them of various appropriate training movements practicable behind the desktop.

##### Physical interventions

**Fig. 3:**
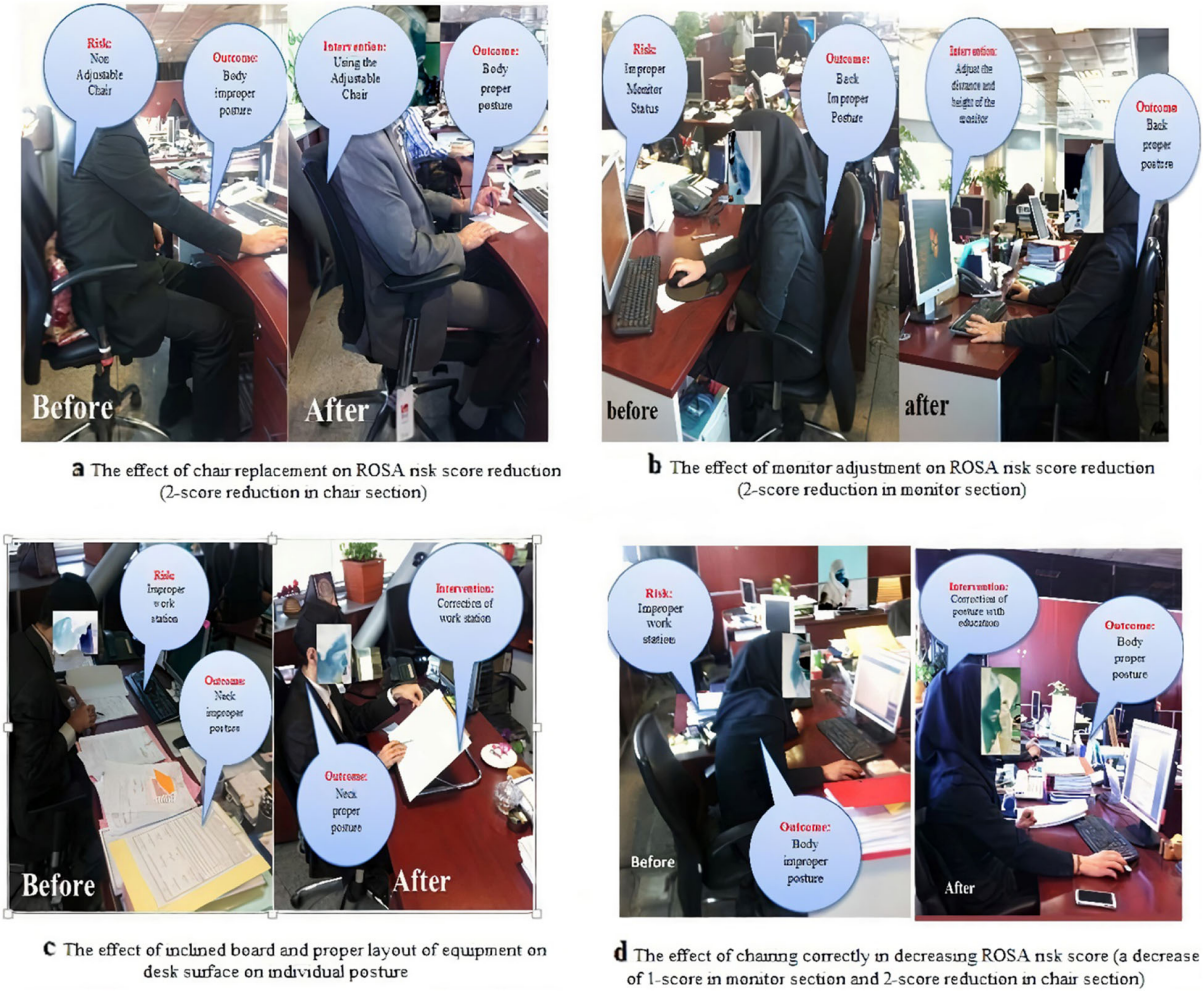
The sample of intervention activities (before and after intervention)

These interventions included the distribution of spine-fit (ergonomic back support pillow); replacement of ergonomic chairs, keyboards, and mouse pads; adjustment of workstations (such as height and angle of the monitor); positioning of the telephone; ergonomic location of work surface equipment; distribution of ergonomic footstools; distribution of inclined boards to raise the height of the study level; and adjustment of the workstation light to prevent glare. These interventions were performed only in the EPI group that required all or part of these interventions according to the ROSA method and its components. Figure 3 presents a sample of intervention activities.

##### Assessing the effectiveness of interventions

Following the intervention, risk factors causing MSDs—and their prevalence—were reassessed through the ROSA method and Nordic questionnaire after 9 months of intervention in the study groups. The effectiveness of ergonomic interventions was determined after the interventions, and the data were compared with those before the interventions.

### Data analysis

SPSS version 16 was used for data analysis. A one-way ANOVA test was used to compare the mean of quantitative variables such as age, work experience, and body mass index (BMI) between the three groups. The McNemar test was used to compare the ratios in qualitative variables such as MSDs of each organ in each group before and after the intervention. A paired *t* test was used to compare the mean score of ROSA and each of its components as a quantitative variable before and after the intervention for all three groups. All tests were conducted at a 95% significance level with *α* = 5%.

## Results

Table 1 summarizes the demographic characteristics of the bank staff in different groups. The results of the one-way ANOVA indicated that the mean of age, BMI, and work experience were similar between the three groups. There was no significant difference between the three groups (*P* > 0.05).

**Table 1:** Demographic characteristics of employees in a large Iranian Bank by study groups

Variable	Group	*n*	Mean	S. deviation	Min	Max	*P* value*
Age (year)	Control group	72	37.23	6.76	24	61	0.068
	EI group	111	36.35	5.52	25	50	
	EPI group	92	34.92	5.45	25	65	
BMI	Control group	72	25.27	4.7	16.53	48.44	0.592
	EI group	109	25.02	3.98	18.13	36.86	
	EPI group	88	26.52	17.55	17.63	180.56	
Work experience (year)	Control group	68	12.75	5.87	3	29	0.136
	EI group	111	12.85	6.35	1	26	
	EPI group	91	11.35	4.6	1	24	

*One-way ANOVA test

Table 2 presents the comparative results of the mean ROSA score (and its components in the workstations of staff) of groups before and after the intervention. There was no significant difference between the mean ROSA score and its components in the control group before and after the intervention. The mean score of ROSA and its components in the EI group decreased significantly following the intervention (*P* < 0.001). The mean score of ROSA and its components in the EPI group were remarkably reduced after the intervention that were statistically significant (*P* < 0.001).

**Table 2:** Comparative results of mean ROSA score and its components in staff workstations of groups before and after intervention

Variable		Control group			EI group			EPI group		
		Before	After	*P* value*	Before	After	*P* value*	Before	After	*P* value*
Chair	Mean	4.41	4.6	0.12	4.63	3.82	< 0.001	4.77	3.47	< 0.001
	S. deviation	0.92	0.81		0.96	0.63		0.87	0.63	
Monitor and telephone	Mean	4.15	4.27	0.083	3.97	1.29	< 0.001	4.18	2.69	< 0.001
	S. deviation	1.32	1.27		1.29	0.59		1.3	0.73	
Mouse and keyboard	Mean	3.8	3.78	0.596	3.38	2.97	< 0.001	3.65	2.77	< 0.001
	S. deviation	1.3	1.22		0.912	0.62		1.07	0.69	
ROSA score	Mean	5.02	5.05	0.673	5.04	3.97	< 0.001	5.3	3.57	< 0.001
	S. deviation	1.02	1.03		0.99	0.59		0.79	0.65	

*Paired sample *t* test

Table 3 presents the comparative results of the prevalence of MSDs in the participants, before and after the intervention. The results of the study of the prevalence of MSDs in the employees before the intervention indicated that during the year, the highest prevalence of MSDs in the control group was in the areas of the neck (67.1%), back (64.4%), and lower back (63%). In the EI group, the highest prevalence was in the neck (65.2%), lower back (61.6%), and back (60.7%) areas. In the EPI group, the discomfort areas were the neck (68.5%), shoulders (66.3%), and lower back (60.9%). The results of the study indicated that (except for the wrist and knee areas) there was a statistically significant difference in the prevalence of MSDs in other areas before and after the intervention in the control group. This difference was due to the increased prevalence of discomfort in these areas (*P* < 0.05). After 9 months of initial evaluation, the incidence of discomfort in all areas of the body, except the wrists and knees, increased in the control group. In the EI group, except for the ankles, the incidence of discomfort was reduced in other areas. However, this decrease was statistically significant only in the neck, shoulder, and lower back regions (*P* < 0.05). The results of the EPI group suggest that—except for the thighs—the prevalence of discomfort was reduced in the other areas. The decrease was statistically significant in the neck, shoulder, wrist, back, and lower back area (*P* < 0.05).

**Table 3:** Comparative results of prevalence of musculoskeletal disorders in different areas of body in three groups before and after intervention

Variable	Control group			EI group			EPI group		
	Before	After	*P* value*	Before	After	*P* value*	Before	After	*P* value*
**Neck**	49 (67.1)	58 (79.5)	0.012	73 (65.2)	50 (44.6)	*< 0.001*	63 (68.5)	40 (34.4)	*< 0.001*
**Shoulders**	44 (60.3)	52 (71.2)	0.021	67 (59.8)	52 (46.4)	*0.04*	61 (66.3)	42 (45.6)	*< 0.021*
**Elbows**	15 (20.5)	25 (34.2)	0.002	21 (18.8)	18 (16.1)	0.375	14 (15.2)	11 (12)	0.375
**Wrist**	38 (52.1)	43 (58.9)	0.18	47 (43)	42 (37.5)	0.12	39 (42.4)	22 (23.9)	*< 0.001*
**back**	47 (64.4)	56 (76.7)	0.012	68 (60.7)	55 (49.1)	*0.061*	54 (58.7)	44 (47.8)	*0.038*
**Lower back**	46 (63)	54 (74)	0.039	69 (61.6)	52 (46.4)	*0.042*	56 (60.9)	26 (28.2)	*< 0.001*
**thighs**	9 (12.3)	19 (26)	0.002	31 (27.7)	20 (17.9)	*0.054*	18 (19.6)	18 (19.2)	*P* > 0.05
**Knees**	40 (54.8)	37 (50.7)	0.549	52 (46.4)	45 (40.1)	0.26	47 (51.1)	40 (43.4)	0.23
**Legs and ankles**	16 (21.9)	22 (30.1)	0.031	21 (18.8)	22 (19.6)	*P* > 0.05	23 (25)	18 (19.5)	0.097

*McNemar’s test

*Significant values are shown in italics

## Discussion

This study aimed to evaluate the risk factors of MSDs using the ROSA method and the implementation of an ergonomic intervention program in an Iranian bank. The results showed that the mean ROSA score and its components decreased following the intervention in the workstations of the EI group and the EPI group. However, there was no difference in the mean score of ROSA, nor its components in the control group workstations that did not receive any intervention during the study. Furthermore, the prevalence of MSDs in several areas of the body of subjects in the EI group and EPI group decreased significantly after 9 months of intervention. However, some of the MSDs had also increased after 9 months of intervention in the control group.

The results of the study of the prevalence of MSDs in the bank employees before the intervention showed that during the year, the highest prevalence of MSDs—in subjects within the control group—was related to the neck, back, and lower back areas. In the EI group, it was in the neck, lower back, and back regions, and in the EPI group it was in the neck, shoulders, and lower back. This prevalence rate is according to the results of previous studies conducted in this area [24–26]. This may be due to the improper design of workstations for these employees. Office work, because of its occupational nature, often requires a static posture and long durations of sitting on a chair—which has been identified as a major risk factor for neck pain, according to recent studies [27]. Prolonged sitting and inappropriate workstation posture can cause long-term static muscle contraction, increased pressure on the intervertebral discs, muscle tension on the ligaments and muscles, and reduced flexibility of tissues and can alter the curvature of the spine. These changes can increase the risk of spinal MSDs [28]. In a study conducted by Mohammadipour et al. (2018) of 250 office workers, the prevalence of musculoskeletal discomfort was high in the staff. The prevalence rate was predominately in the lower back and neck [24]. Besharati et al. (2020) similarly highlighted that the prevalence of these disorders among office workers in the neck and shoulder areas was higher than in other areas. This is consistent with the results of the present study [25]. In another study conducted by Giahi et al. (2014), to examine MSDs in computer users of bank office staff, the highest prevalence was reported in the neck, lower back, elbows, and thighs [19].

Studying risk factors causing MSDs preintervention suggested that the mean score of ROSA in workstations of all three groups was above five (high-risk level). However, the mean final score of this method—and its components in the intervention groups—showed a significant decrease following the intervention compared with the time when no intervention was undertaken. However, the mean of this method (and its components) did not change in the control group that did not receive the intervention. These findings indicate the impact of EPIs on reducing ergonomic risk levels in workstations. The lack of employees’ awareness of the benefits of ergonomics before doing interventions and the use of non-ergonomic equipment—and their insufficient attention to ergonomic issues in the workplace—may be the main reasons for high workstation ergonomic risk score before the intervention. However, increased awareness of employees through education, ergonomic workstation design, physical changes in the workstations and replacement of some non-ergonomic equipment reduced the level of risk to acceptable levels. Poochada et al. (2015) investigated ergonomic risk factors among office workers within telephone centers using the ROSA method. The findings suggested that the majority of people were at a high risk level (above five), and therefore, rapid workstation modification was required [29]. Additionally, the mean ROSA score and its components in the present study are higher than those obtained in the study of Sonne et al. (2012). This indicates a worse condition of workstations in present study compared with Sonne et al. and generally confirms the existence of high risk levels in office settings [30]. Shariat et al. (2018) found that the use of ergonomic modification—along with the use of stretching exercises—can significantly improve the status of workstations and MSDs prevalence in office workers [31]. The results of similar studies also confirm the positive effect of EPIs on reducing the level of ergonomic risk factors in office work environments. These findings are according to the results of the present study [32].

The results of the present study showed that except for the ankles, the prevalence of disorders in other areas of the body decreased. However, this decrease was statistically significant only in the neck, shoulder, and lower back areas. Ergonomics training is a tool that can be used to increase employee knowledge of how to adjust the layout of work, maintain the most beneficial postures while performing work, correctly use office equipment, and adjust chair and monitor heights. Consequently, the workstation’s ergonomic status can be improved by employees [33]. In this study, after enhancing users’ knowledge—by distributing training handbooks and helping them adjust workstations by themselves during the study period—there was a significant improvement in working postures, monitor height, telephone usage, and chair adjustment. The decrease in the ROSA score following the intervention also confirms these findings. Consequently, these improved conditions could, after a period of 9 months, cause a significant decrease in the prevalence of MSDs in various areas of the body. These results are according to the results of studies by Robertson et al. (2009), Zeidi et al. (2011), Mahmud et al. (2011), and Motamedzade et al. (2011). These studies all used ergonomics training to improve the ergonomic conditions of workstations and reduce the prevalence of MSDs in computer users and office workers [33–36]. These results are in contrast to a study conducted by Ali Arabian et al. (2013) who did not find EI alone to be a suitable method for reducing MSDs [37].

The results of the study of the prevalence of MSDs in the EPI group—before the intervention and 9 months after the beginning of the intervention—showed that except in the thighs, the prevalence of other disorders decreased. These findings suggest that implementing ergonomic PIs (replacing chairs and using ergonomic chairs, changing workstation layouts, and replacing a non-ergonomic mouse, providing a sheet holder, offering a mouse pad, adjusting the height of the monitor) along with administrative intervention (teaching people how to maintain proper posture while working, how to use the mouse and keyboard correctly, how to work with the phone correctly, adjusting the chair, using breaks during work, and doing soft movements behind the desk) has been able to significantly reduce MSDs in several areas of the body. Furthermore, the rate of decline, compared with the group receiving the education program, was greater. One of the most important reasons for the higher effectiveness of these interventions, compared with the EI group, can be the combined use of PIs with EIs. This approach is considered to be the most important and effective measure to improve ergonomic conditions in workplaces [22]. These results are consistent with studies, in which PIs have been used in addition to administrative (educational) interventions. In a study of four groups of computer users and office workers, Robertson et al. (2009) indicated that after 6 months of intervention, the reduction level in the prevalence of discomfort in the group receiving both physical and administrative interventions was higher than in the other three groups (control group, the EPI group, and the PI group) [36]. In the study conducted by Jahangiri et al. (2015), both PIs and EIs were introduced as a more appropriate approach than the use of administrative intervention (employee training) alone to reduce musculoskeletal discomfort [22]. The findings of studies conducted by Shariat et al. (2018) and Robertson et al. (2016) are consistent with the results of the present study [31, 32].

### Study limitations

One of the strengths of the present study was its large sample size. However, conducting this study with this high number of participants required a large amount of time and financial resources. It was conducted with the support of the supervisors of the study units. Additionally, the management commitment (unit supervisors) required to support the study, and the high participation of the surveyed staff may be one of the important factors in achieving results. The use of a specific method (ROSA) to assess risk factors causing MSDs in office occupations that incorporate many of the biomechanical risk factors associated with such occupations is another strength of the present study. One of the weaknesses of the present study is studying the prevalence of MSDs in the population using a self-reported questionnaire (Nordic musculoskeletal questionnaire), because this method may be affected by the subjects [23]. Finally, the results of the present study indicate the increased effect of both EPIs compared with EIs alone and confirm the use of the ROSA method in determining the type of interventions required.

## Conclusions

Nine months after interventions, relative improvement in workstations and prevalence of MSDs was observed in the intervention groups. These findings suggest that implementing educational and physical intervention ergonomic will further reduce MSDs compared to educational intervention alone. Moreover, increasing employees’ awareness of the ergonomic risk factors in office work, and the correct way to arrange and utilize office equipment can improve the workstation—through the employees themselves—and thus improve conditions.

## Data Availability

The datasets generated during the current study are available on request.
